# Interaction in Assistive Robotics: A Radical Constructivist Design Framework

**DOI:** 10.3389/fnbot.2021.675657

**Published:** 2021-06-09

**Authors:** Marco C. Bettoni, Claudio Castellini

**Affiliations:** ^1^Steinbeis Consulting Centre, Knowledge Management and Collaboration (KMC), Basel, Switzerland; ^2^The Adaptive Bio-Interfaces Group, German Aerospace Centre (DLR), Institute of Robotics and Mechatronics, Oberpfaffenhofen, Germany

**Keywords:** upper-limb prosthetics, myocontrol, machine learning, incremental learning, human-robot interaction, human-machine interfaces, radical constructivism, interaction design

## Abstract

Despite decades of research, muscle-based control of assistive devices (myocontrol) is still unreliable; for instance upper-limb prostheses, each year more and more dexterous and human-like, still provide hardly enough functionality to justify their cost and the effort required to use them. In order to try and close this gap, we propose to shift the goal of myocontrol from guessing intended movements to *creating new circular reactions* in the constructivist sense defined by Piaget. To this aim, the myocontrol system must be able to acquire new knowledge and forget past one, and knowledge acquisition/forgetting must happen *on demand*, requested either by the user or by the system itself. We propose a unifying framework based upon Radical Constructivism for the design of such a myocontrol system, including its user interface and user-device interaction strategy.

## Introduction

According to the layman's definition, a Human-Machine Interface (HMI) is the hardware/software system enabling a user control a device (computer, robot, tool, etc.); it is the channel through which user-device *interaction* takes place (Castellini, 2016). Unsurprisingly, most HMIs rely on the assumption that the user can voluntarily and precisely control arms, hands and fingers—think, e.g., the handles of a wheelbarrow, the cockpit of an airplane, and the surface and operating system of a smartphone. However, this assumption fails when the device to be controlled is an assistive one. An upper-limb amputee using a prosthetic arm in daily life or a stroke survivor progressively getting in control of a rehab exoskeleton cannot properly use their limbs to control their machines—here a more flexible and smart kind of HMI is required (Beckerle et al., 2018), able to interpret the user's intent to move using bodily signals typically related to muscle activation (*myocontrol*, see, e.g., Castellini et al., 2014).

But myocontrol is still unreliable, notwithstanding three decades of intense research (Schweitzer et al., 2018). The human-friendliness and dexterity of upper-limb prostheses, for instance, increases every year, while their rejection rate remains high, largely due to poor myocontrol (Vujaklija et al., 2016). Better sensors, better physical interfaces and better machine-learning (ML) methods and models are the main avenues researchers are pursuing (Fougner et al., 2012; Jiang et al., 2012); still, without neglecting these issues, a fundamental ingredient the recipe lacks is a tight *coupling* between user and machine (Hahne et al., 2017; Beckerle et al., 2018). Coupling arises from reciprocal adaptation which in turn relies on “transparent” control of the device—the device should move according to the user's wishes without the user even consciously realizing it (Makin et al., 2017).

Here, a somewhat deeper psychological interpretation of the informal notion of transparent control (Fougner et al., 2012) is required. Musculoskeletal impairments, preventing motor commands from being correctly executed, lead to the disappearance of *circular reactions*—basic sensori-motor associations created during the infancy by interacting with the environment (Piaget, 1966; Evans, 1973; Sanchez and Loredo, 2007), significantly degrading the patient's quality of life. But they can also be restored/created anew by exploiting the plasticity of the neural circuitry which can be induced, e.g., in virtual reality (Yanagisawa et al., 2016), sometimes with consequences on the perception of pain. Myocontrol could possibly then be used to foster the restoration, or novel creation, of such circular reactions, to replace those destroyed by the patient's condition. Correct and reliable intent interpretation would then be a desirable side effect.

To this aim, the ideal assistive device reacts as dexterously and quickly as the musculoskeletal system itself (Botvinick and Cohen, 1998) while providing proper sensory feedback in real time; but this is just a necessary condition. The user must also be involved in a fruitful sensorimotor interaction with the device, *teaching* it how it should work (Nowak et al., 2018). We believe that a Radical-Constructivist (von Glasersfeld, 1995) framework can unify all these aspects and provide useful guidelines for the design of better ML systems, user interfaces and experimental protocols for myocontrolled assistive devices.

## On the Purpose of Assistive Systems

In mammals (actually, in all beings endowed with a nervous system) every single movement produces a “sensorial trace”—in the simplest setting, indeed a proprioceptive one. Simple, basic, stereotyped movements corresponding to similarly simple sensorial traces, for instance the act of flexing a wrist and the feeling of flexing it, become strongly *associated* to each other through repeated execution since birth, thanks to the plasticity of the nervous system. According to Piaget, such sensori-motor associations are the building blocks of one's own body control and even, possibly, of intelligence *tout court* (Piaget, 1966); the paradigm of enactive/embodied knowledge and learning points in the same direction (de Bruin et al., 2018).

### Radical Constructivism

Piaget's theory of cognitive development contends that, in the 1st month after birth, an infant's activity is characterized by simple, genetically determined reflexes such as rooting and sucking; subsequently, till 4 months of age, the interest shifts to the body, trying to *reproduce* pleasant events—a rudimentary form of goal-directedness (Piaget, 1966). These cyclic behaviors had been called *circular reactions* by Baldwin (Baldwin, 1894) because a random action would generate a pleasant stimuli leading to the repetition of said action. Piaget further developed this idea by introducing the concepts of *assimilation, accommodation, organisation* and *action scheme*, which led him to further distinguish primary, secondary (4–12 months) and tertiary (12–18 months) circular reactions (Piaget, 1966).

In particular, an action scheme (von Glasersfeld, 1995) is a goal-directed extension to the traditional stimulus-response reflex model, consisting of (1) the recognition of a specific situation; (2) the execution of an action associated with that situation; and (3) the comparison of the new situation, obtained as a consequence of the action, to an expected (desired) result. The infant will first recognize a situation as an instance of something known (assimilation, Piaget, 1966; von Glasersfeld, 1995), then it will execute an activity associated with it, and lastly, it will try to assimilate the obtained result to its expectations. If this attempt fails, either the initial recognition will be modified, in order to prevent further triggering of the same action in the future, or a new scheme will be created, by modification of the expected result (accommodation).

The continual execution of action schemes, at first at random, then in a progressively coordinated fashion, leads to their self-organisation into more and more complex ones, effectively building up sensorimotor coordination in the infant. In order to form, use and organize action schemes, however, an infant needs a set of basic capabilities, namely (von Glasersfeld, 1995) to be able (a) to *remember* and *retrieve* past experiences; (b) to *compare* and *determine (dis)similarity* between them and the current situation; and (c) to *evaluate* experiences as interesting and/or beneficial, that is, to match them against a goal. The need for such a system-oriented perspective leads us to adopt a more operational kind of constructivism then Piaget's, *Radical Constructivism* (von Glasersfeld, 1995). RC is based upon six fundamental principles, derived more in detail from Piaget (1966), von Glasersfeld (1983, 1995), Varela et al. (1991), Kant (1998):

1. *[Experiential world]* Although all human beings share the same physical space, each one lives in a secluded experiential world, an inner universe constructed by interacting with the environment.
2. *[Objects]* The objects found in the experiential world *use* the environment but are not determined by/do not conform to it; rather, they are determined by/conform to the way the individual constructs them. This idea goes back to Immanuel Kant's *Copernican revolution* (Kant, 1998).
3. *[Functions]* Objects are constructed via a self-organizing system of *basic functions*: reflexes, circular reactions, assimilation, accommodation and organisation (Piaget, 1966).
4. *[Autopoiesis]* This self-organizing system is autopoietic (Varela et al., 1991): the outcomes of the construction extend and further develop the basic functions that constructed them.
5. *[Viability]* In the process of constructing the experiential world, *viable* objects are preferred—objects which better fulfill the goal for which they have been constructed (von Glasersfeld, 1983).
6. The *[environment]* then provides material for the construction of each individual's experiential world and puts to the test its viability.

### Musculoskeletal Impairments From the RC Perspective

Primary and secondary circular reactions clearly encompass the above-mentioned intuitive notion of (simple, basic, stereotyped) “sensorimotor associations.” They are subsequently hierarchically organized in action schemes thanks to assimilation, accommodation and organisation. Action schemes corresponding to more complex, high-level, goal-directed actions can be decomposed into finer-grained action schemes, and, in the end, into their constituent circular reactions (this idea already appears in, e.g., von Glasersfeld, 1995; Kumar et al., 2018). For instance, “reaching for and grasping a cup of tea” can be decomposed into simpler actions schemes, e.g., “focus on the cup,” “stretch the arm,” “pre-shape the hand,” etc. Each such scheme can be decomposed in turn, till primary and secondary circular reactions are reached. Each time such an action scheme is executed, all lower-level action schemes and circular reactions it involves are executed in turn. This way, the organisation of the objects at the core of this action continually consolidates and adapts, increasing its own viability and tightening the coupling between the environment and the subject.

Seen from this perspective, acquired musculoskeletal impairments disrupt specific sets of primary and/or secondary circular reactions, and, consequently, all action schemes based upon them. As a consequence, the organization of these schemes gets gradually undone. A trans-radial amputation, for example, annihilates—among others—all secondary circular reactions related to the missing wrist, as well as all higher-level ones based upon them. Phantom-limb sensation and pain and maladaptive cortical reorganization (Flor and Birbaumer, 2000; Erlenwein et al., 2021) can probably be seen as consequences of such a disruption. In RC terms, the experiential world of those who suffer from a musculoskeletal impairment undergoes a dramatic reorganisation; objects which had been constructed during the patient's life as a healthy person disappear and new action schemes are constructed, which are necessarily much less viable then before. Following the previous example, trans-radial amputees shift the dominance to the remaining limb, adapt the gait to the altered weight of the body and perform manipulation tasks using compensation movements (Schweitzer et al., 2018)—these are only some of the new experiential objects they construct. An amputation significantly reduces the patient's quality of life; nevertheless, the new action schemes are the most viable *given the prosthetic system at the patient's disposal* and the autopoietic nature of the objects in the patient's experiential world.

### Myocontrol as a Means to Fix a Broken Experiential World

A prosthetic system (the prosthetic device plus its myocontrol system) should then aim at bi-directionally connecting the patient to the device in such a tight way that novel primary and secondary circular reactions form, functionally replacing the missing ones and constituting the basis of new action schemes; this would translate to better feeling of immersion and embodiment, more trust in the prosthesis, better control and higher functionality in daily living. These ideas, moreover, apply to all assistive devices requiring fine control by a disabled user (exo-suits, exoskeleta for rehabilitation, active orthoses, virtual rehabilitation systems, etc.) via residual muscle activity—wherever myocontrol is involved.

We contend that the ideal assistive system should foster the re-organisation of the patient's experiential world, rather than detecting the patient's intent. For instance, it should enable an amputee flex and extend the wrist with such a short latency and high precision, that no *conscious* attempt to do it is felt; and it should provide such an apt and subtle substitute feeling for the flexion/extension of the wrist, that the association between the action and the feeling becomes intimate, indissoluble—indeed, a new primary circular reaction. Currently, no prosthetic device is able to provide such a swift motion, but virtual reality is a viable test-bed, for instance to ease neuropathic pain or as a prosthetic training environment (Ortiz-Catalán et al., 2014; Nissler et al., 2019). Such a claim is substantiated by numerous hints found in literature about the swiftness of self-powered prostheses and its fallout on prosthetic rejection, for instance relating the feeling of immersion and embodiment to short mechanical latency, its looks and the reliability of myocontrol (Farrell and Weir, 2007; Smith et al., 2011; Beckerle et al., 2018).

An RC myocontrol system is then a bidirectional interface (Beckerle et al., 2018) translating actions and feelings back and forth, fostering the construction of new circular reactions.

## Interaction for Assistive Robotics: A RC Perspective

In the previous Section we have tried to provide the RC perspective on musculoskeletal impairments and the aim of RC myocontrol. We now sketch its characteristics and give an example of it.

### Radical-Constructivist Myocontrol

Consider the six principles mentioned above, which RC-based myocontrol should adhere to. Its *experiential world* is the space of signals available to interact with the user, typically, bio-signals gathered from the user and environmental signals provided by the device and the physical environment. The *objects* in its experiential world, constructed in the course of time, are (a) signal patterns gathered from the user while trying to perform specific actions; and (b) a model associating signal patterns to said actions. *Assimilation* and *accommodation* correspond then to defining the patterns/actions associations in the model: a pattern can be associated to an existing action (assimilation), or associated to a new action or rejected (accommodation). Assimilation and accommodation are therefore *supervised* functions—the system needs to interact with one or more oracles to know the pattern/action association, e.g., the experimenter, the user or a decision procedure.

Finally, the *organisation* of the objects in the system's experiential world corresponds to the creation or adjustment of the above-mentioned model, determining its *viability*—the degree to which the predicted actions adhere to the patient's desires and needs. In a virtuous loop of data acquisition and reorganisation of the objects, the viability increases in time, possibly reaching a local optimum.

Operationally—consider again the three basic requirements of a constructivist system highlighted above—RC myocontrol must be able to

1. match a signal pattern to a previous one, estimating the confidence of the match;
2. store, delete, retrieve and forget signal patterns; and
3. decide to acquire new patterns and/or ask the user to provide new data, or delete past patterns.

### Flexing a Virtual Wrist, in a Radically-Constructivist Sense

How would a typical RC myocontrol look like, in practice? We believe that it would consist of a *(supervised) incremental machine learning method, updated on-demand via carefully designed interaction with its oracles*. More in detail, basic requirements (1) and (2) are provided already by standard, supervised machine learning, where building a pattern/action model is enforced by minimizing a cost functional. Furthermore, *interactive* machine learning provides the ability to gather and assign new patterns/delete past ones and update the model given the new set of patterns. Item (3) can be enforced by querying the user and/or the experimenter/therapist by using, e.g., *measures of confidence* of a pattern match. An initial attempt can be seen in Nowak et al. (2018).

The interaction with a human oracle, however, seems more problematic; here, specific attention must be given to the interaction protocols and to the interface to the user and/or the experimenter, which must be readily, intuitively *interpretable*, allowing the user to form a suitable *mental model* of the device. The principles of Interaction Design (IxD, Norman, 2013), a branch of Design Science concerned with usability and friendliness of devices, could help. The main predicament of IxD is that objects should be designed such that they can be used in the right way only (“human errors are design errors”). In the case of myocontrol, there should be one way only to teach the device which patterns correspond to which signals, and to have it acquire and forget data (from this perspective most of IxD seems based upon constructivist principles).

Following up the previous example of the wrist flexion, we now sketch a possible RC wrist myocontrol system. The system's objects are two signal patterns, one for the resting state and one for the full wrist flexion, gathered in the course of time from a patient using surface electromyography (Merletti et al., 2011). A regression method has been used to build a proportional model of the wrist flexion, and a realistic virtual reality wrist closely and swiftly displays the estimated wrist flexion to the patient. Electro-cutaneous stimulators (see, e.g., González-Vargas et al., 2015) are used to convey a feeling of proportional intensity to the patent's forearm. Within the limits of the virtual world, the patient can walk and freely move the arm and forearm while flexing the wrist. Each time the wrist flexion does not reflect the patient's desire, for instance because of the limb-position effect (Campbell et al., 2020), the patient can act on either of two virtual buttons on the forearm, clearly labeled with a resting wrist and a fully flexed one. Pressing one of the buttons starts a further data gathering related to the action represented on the button itself; the model is instantaneously updated.

A further element of the user interface is sound feedback, issued whenever the confidence of the model estimation drops below a threshold; this feedback denotes the necessity for the patient to provide more data in an area of the input space where the uncertainty is high [this strategy already appears in Gigli et al. (2020)]. In the course of the time spent within the virtual world, we expect the objects in the experiential world of the myocontrol system, that is the signal patterns corresponding to the resting state/full flexion and the regression model, to increase their viability with respect to the environment, and a new hierarchy of circular reactions related to the flexion of the wrist to arise in the user's experiential world. As a side-effect, the enaction of the virtual wrist flexion becomes more and more accurate with respect to the patient's desire—this can easily be assessed by administering a TAC test (Simon et al., 2011) to the patient at specific intervals of time.

## Concluding Remarks

The requirement that the user be at the centre of research in assistive robotics is nowadays relevant in literature and is clear from the growing number of research projects in which clinics and healthcare companies are involved. User-centred design should be employed at all stages, and this requires a deeper understanding of the neurological and psychological processes behind (re)learning new sensorimotor faculties. In this perspective article we have argued that Radical Constructivism offers such a theoretical and practical framework to conceive and design a human-centered approach to assistive robotics; in particular, that RC can be used to understand musculoskeletal impairments, shift the paradigm of myocontrol and set a new aim to it, and design in a principled way the HMI and the patient-device interaction.

A number of open issues remain, three of which seem particularly interesting at the time of writing. In the first place, interactive machine learning has been explored only marginally so far in myocontrol, the classical approach being the collection of data at the start of each control session (Castellini, 2016), therefore there is yet no comparison. The potential superiority of one approach with respect to the other will be proven only in the course of time and via testing on end-users. Secondly, we are aware of no neural correlates of circular reactions that can be detected with state-of-the-art brain imaging techniques (although, e.g., Virji-Babul et al., 2012 is a promising study going in this direction), so how to detect the creation and disappearance of novel circular reactions induced by RC myocontrol is still an open question. Lastly, a way of numerically determining the interpretability of an assistive system is a fascinating, although still unexplored, issue.

## Data Availability Statement

The original contributions presented in the study are included in the article/supplementary material, further inquiries can be directed to the corresponding author/s.

## Author Contributions

All authors listed have made a substantial, direct and intellectual contribution to the work, and approved it for publication.

## Conflict of Interest

The authors declare that the research was conducted in the absence of any commercial or financial relationships that could be construed as a potential conflict of interest.

## References

[B1] Baldwin J. M. (1894). Imitation: a chapter in the natural history of consciousness. Mind 3, 26–55. 10.1093/mind/III.9.26

[B2] Beckerle P. Castellini C. Lenggenhager B. (2018). Robotic interfaces for cognitive psychology and embodiment research: a research roadmap. Wiley Interdisc. Rev. 10:e1486. 10.1002/wcs.1486 30485732

[B3] Botvinick M. Cohen J. (1998). Rubber hands “feel” touch that eyes see. Nature 391:756. 10.1038/35784 9486643

[B4] Campbell E. Phinyomark A. Scheme E. (2020). Current trends and confounding factors in myoelectric control: limb position and contraction intensity. Sensors 20:1613. 10.3390/s20061613 32183215 PMC7146367

[B5] Castellini C. (2016). Incremental learning of muscle synergies: from calibration to interaction, in Human and Robot Hands: Sensorimotor Synergies to Bridge the Gap Between Neuroscience and Robotics, eds Bianchi M. Moscatelli A. (Springer International Publishing). 10.1007/978-3-319-26706-7_11

[B6] Castellini C. Artemiadis P. Wininger M. Ajoudani A. Alimusaj M. Bicchi A.. (2014). Proceedings of the first workshop on peripheral machine interfaces: going beyond traditional surface electromyography. Front. Neurorobot. 8:22. 10.3389/fnbot.2014.00022 25177292 PMC4133701

[B7] de Bruin L. Gallagher S. Newen A. (Eds.). (2018). The Oxford Handbook of 4E Cognition. Oxford: The Oxford University Press.

[B8] Erlenwein J. Diers M. Ernst J. Schulz F. Petzke F. (2021). Clinical updates on phantom limb pain. PAIN Rep. 1:e888. 10.1097/PR9.0000000000000888 33490849 PMC7813551

[B9] Evans R. I. (1973). Jean Piaget, the Man and his Ideas. New York, NY: E. P. Dutton & Co.

[B10] Farrell T. R. Weir R. F. (2007). The optimal controller delay for myoelectric prostheses. IEEE Trans. Neural. Syst. Rehabil. Eng. 15, 111–118. 10.1109/TNSRE.2007.891391 17436883 PMC8173529

[B11] Flor H. Birbaumer N. (2000). Phantom limb pain: cortical plasticity and novel therapeutic approaches. Curr. Opin. Anaesthesiol. 5, 561–564. 10.1097/00001503-200010000-00013 17016358

[B12] Fougner A. Stavdahl Ø. Kyberd P. J. Losier Y. G. Parker P. A. (2012). Control of upper limb prostheses: terminology and proportional myoelectric control - a review. IEEE Trans. Neur. Syst. Rehab. Eng. 20, 663–677. 10.1109/TNSRE.2012.2196711 22665514

[B13] Gigli A. Brusamento D. Meattini R. Melchiorri C. Castellini C. (2020). Feedback-aided data acquisition improves myoelectric control of a prosthetic hand. J. Neural. Eng. 17:056047. 10.1088/1741-2552/abbed0 33022665

[B14] González-Vargas J. Dosen S. Amsüss S. Yu W. Farina D. (2015). Human-machine interface for the control of multi-function systems based on electrocutaneous menu: application to multi-grasp prosthetic hands. PLoS ONE 10, 1–26. 10.1371/journal.pone.0127528 26069961 PMC4466571

[B15] Hahne J. M. Markovic M. Farina D. (2017). User adaptation in myoelectric man-machine interfaces. Sci. Rep. 7:4437. 10.1038/s41598-017-04255-x 28667260 PMC5493618

[B16] Jiang N. Dosen S. Müller K.-R. Farina D. (2012). Myoelectric control of artificial limbs - is there a need to change focus? IEEE Signal. Process. Magazine 29, 148–152. 10.1109/MSP.2012.2203480

[B17] Kant I. (1998). Critique of Pure Reason. New York, NY: Cambridge University Press. 10.1017/CBO9780511804649

[B18] Kumar S. Shaw P. Giagkos A. Braud R. Lee M. Shen Q. (2018). Developing hierarchical schemas and building schema chains through practice play behavior. Front. Neurorobot. 12:33. 10.3389/fnbot.2018.00033 29988610 PMC6027137

[B19] Makin T. R. de Vignemont F. Faisal A. A. (2017). Neurocognitive barriers to the embodiment of technology. Nat. Biomed. Eng. 1:14. 10.1038/s41551-016-0014

[B20] Merletti R. Aventaggiato M. Botter A. Holobar A. Marateb H. Vieira T. M. (2011). Advances in surface EMG: recent progress in detection and processing techniques. Crit. Rev. Biomed. Eng. 38, 305–345. 10.1615/CritRevBiomedEng.v38.i4.10 21133837

[B21] Nissler C. Nowak M. Connan M. Büttner S. Vogel J. Kossyk I.. (2019). VITA - An everyday virtual reality setup for prosthetics and upper-limb rehabilitation. J. Neural Eng. 16:026039. 10.1088/1741-2552/aaf35f 30864550

[B22] Norman D. A. (2013). The Design of Everyday Things (2 ed.). New York, NY: Basic Books.

[B23] Nowak M. Castellini C. Massironi C. (2018). Applying radical constructivism to machine learning: a pilot study in assistive robotics. Construct. Found. 13, 250–262. Available online at: http://constructivist.info/13/2/250.nowak

[B24] Ortiz-Catalán M. Sander N. Kristoffersen M. B. Håkansson B. Brånemark R. (2014). Treatment of phantom limb pain (PLP) based on augmented reality and gaming controlled by myoelectric pattern recognition: a case study of a chronic PLP patient. Front. Neurosci. 8:24. 10.3389/fnins.2014.00024 24616655 PMC3935120

[B25] Piaget J. (1966). The Origins of Intelligence in Children; Translated by Margaret Cook. New York, NY: International Universities Press.

[B26] Sanchez J. C. Loredo J. C. (2007). In circles we go: Baldwin's theory of organic selection and its current uses: a constructivist view. Theory Psychol. 17, 33–58. 10.1177/0959354307073150

[B27] Schweitzer W. Thali M. J. Egger D. (2018). Case-study of a user-driven prosthetic arm design: bionic hand versus customized body-powered technology in a highly demanding work environment. J. NeuroEng. Rehabilit. 15:1. 10.1186/s12984-017-0340-0 29298708 PMC5751817

[B28] Simon A. M. Hargrove L. J. Lock B. A. Kuiken T. A. (2011). Target Achievement Control Test: Evaluating real-time myoelectric pattern recognition control of multifunctional upper-limb prostheses. J. Rehabilit. Res. Dev. 48, 619–628. 10.1682/JRRD.2010.08.0149 21938650 PMC4232230

[B29] Smith L. H. Hargrove L. J. Lock B. A. Kuiken T. A. (2011). Determining the optimal window length for pattern recognition-based myoelectric control: balancing the competing effects of classification error and controller delay. IEEE Trans. Neural. Syst. Rehabil. Eng. 19, 186–192. 10.1109/TNSRE.2010.2100828 21193383 PMC4241762

[B30] Varela F. G. Maturana H. R. Uribe R. (1991). Autopoiesis: the organization of living systems, its characterization and a model, in Facets of Systems Science (Boston, MA: Springer US). 10.1007/978-1-4899-0718-9_40 4407425

[B31] Virji-Babul N. Rose A. Moiseeva N. Makan N. (2012). Neural correlates of action understanding in infants: influence of motor experience. Brain Behav. 2, 237–242. 10.1002/brb3.50 22741097 PMC3381628

[B32] von Glasersfeld E. (1983). Learning as constructive activity, in Proceedings of the 5th Annual Meeting of the North American Group of Psychology in Mathematics Education (Montreal, QC: PME-NA), Vol. 1, 41–101. Available online at: http://vonglasersfeld.com/083

[B33] von Glasersfeld E. (1995). Radical Constructivism: A Way of Knowing and Learning. London: Falmer Press.

[B34] Vujaklija I. Farina D. Aszmann O. (2016). New developments in prosthetic arm systems. Orthopedic Res. Rev. 8, 31–39. 10.2147/ORR.S71468 30774468 PMC6209370

[B35] Yanagisawa T. Fukuma R. Seymour B. Hosomi K. Kishima H. Shimizu T.. (2016). Induced sensorimotor brain plasticity controls pain in phantom limb patients. Nat. Commun. 7:13209. 10.1038/ncomms13209 27807349 PMC5095287

